# Kinetic Assessment of Kraft and Thermally Upgraded Kraft Papers Aged in Five Alternative Dielectric Fluids

**DOI:** 10.3390/polym16091196

**Published:** 2024-04-25

**Authors:** Cristina Méndez, Cristian Olmo, Carlos Javier Renedo, Alfredo Ortiz, Diego García

**Affiliations:** 1 Electrical and Energy Engineering Department, School of Industrial and Telecommunications Engineering, Universidad de Cantabria, Avenida Los Castros, 39005 Santander, Spain; cristian.olmo@unican.es (C.O.); carlos.renedo@unican.es (C.J.R.); alfredo.ortiz@unican.es (A.O.); 2 School of Electrical and Electronic Engineering, Universidad del Valle, Cali 760032, Colombia; diego.garcia@correounivalle.edu.co

**Keywords:** cellulose, polymerisation degree, degradation, kinetic model, insulating fluids

## Abstract

The lifespan of an electrical transformer, primarily determined by the condition of its solid insulation, is well known under various operating conditions when mineral oil is the coolant in these machines. However, there is a trend toward replacing this oil with biodegradable fluids, especially esters; therefore, an understanding of the ageing of solid insulation with these fluids is essential. Currently available data do not allow for the selection of the best ester among those available on the market, as each study applies different conditions, making it impossible to compare results. Thus, this paper analyses the degradation of Kraft and Thermally Upgraded Kraft papers with the following five most promising commercial esters: sunflower, rapeseed, soybean, palm, and synthetic. The materials underwent accelerated thermal ageing at 130, 150, and 170 °C, and the integrity of the papers was evaluated through their polymerisation degree and the obtaining of the degradation kinetic models. The wide range of materials studied in this work, which were subjected to the same treatments, allows for a comparison of the esters, revealing significant differences in the impact of the alternative fluids. Sunflower, rapeseed, and soybean esters provided the best paper protection, i.e., the degree of polymerisation of Kraft paper in the tests at 150 °C decreased by 71% with these fluids, compared to the 83% reduction with mineral oil, 79% reduction with palm ester, and 75% reduction with synthetic ester. Furthermore, different kinetic models were obtained to predict the degradation; it was concluded that the Emsley model provides the best fit. Additionally, it was found that the behaviour of a dielectric fluid with one type of paper cannot be extrapolated, which is only noticeable in broad-scope studies.

## 1. Introduction

Despite the efficiency of oil-based cooling systems in power transformers, losses in the wires lead to increased temperatures, causing long-term damage [1]. Recurrent high temperatures particularly affect the solid insulation of the wires, primarily composed of cellulose polymers, whose chain integrity defines the equipment’s lifespan [2]. The polymers break by the following three main mechanisms, depending on the environmental conditions: hydrolysis, oxidation or pyrolysis [3]. 

In recent years, the substitution of the predominant mineral oils in transformers [4,5] with eco-friendly alternatives [6] as cooling fluids has been promoted. This applies to both new equipment and the retrofilling of existing transformers, refs. [7,8], to extend their lifespan and minimize environmental impacts. Currently, natural and synthetic esters are the most widespread options due to their biodegradability and performance capabilities [9,10]. However, it is not as simple as just replacing the oils, since the degradation of cellulose depends not only on its composition and treatment, but also on the dielectric fluid with which it is combined, refs. [11,12,13]. For this, reliable degradation data of the polymer with the alternative materials are fundamental.

Given the essential role of transformers in the electrical system and their cost [14], predicting the remaining life of their components is critical to avoid severe disruptions in electrical supply [15,16,17]. Various methodologies and techniques have been developed to predict transformer component life accurately based on operational data [18,19,20]. Nevertheless, when introducing new materials, there is a lack of available data from operational equipment, and laboratory tests are necessary to determine the feasibility of using these alternatives. These tests often involve measuring the time evolution of the polymerisation degree (DP) of cellulose or the mechanical properties of transformer paper samples under thermal stress [21,22,23]. Esters differ chemically from mineral oils and from each other, depending on the fatty acids and alcohols from which they are derived [9,24]. Consequently, experimental results may reflect this variability, allowing for the identification of the best alternative.

While numerous aging studies on esters have shown positive effects, their diverse methodologies hinder finding the best alternative fluid. More than 90% of these studies analyse only one or two esters, refs. [9,13,22,25,26], and there are variations in methodologies and in the conditions applied. For example, the test temperature can range from as low as 70 °C, ref. [27], to as high as 250 °C [28], affecting the degradation rate and the mechanisms involved [3]. Similar variations occur with the atmosphere in which the ageing process takes place [29], and the materials considered in the test, ref. [26]. Even quite similar experiments by different research groups yield non-comparable results, ref. [30]. These differences affect the development of useful polymer degradation prediction models for these fluids, with activation energy values as diverse as 86.94 kJ/mol in one study, ref. [31], and 120.64 kJ/mol in another, ref. [32].

Given these challenges and the available knowledge, it is necessary to analyse the behaviour of a wider variety of fluids and types of paper under thermal stress, consistently applying the same methodology to ensure comparable data. This work not only presents the evolution of the DP and how it depends on several factors, but also a study of kinetic models for predicting thermal degradation. It has been carried out at three ageing temperatures, analysing two types of transformer paper combined with the top five commercial dielectric esters, for a total of 36 combinations. The focus of the study is on dispelling any remaining doubts about the application of esters in transformers.

## 2. Materials and Methods

This section describes the materials studied in this work and the procedure followed to treat and analyse the samples.

### 2.1. Materials

The insulation systems for this work are composed of insulating paper and dielectric fluid. Two commercial dielectric papers were analysed; a medium-density Kraft paper and a thermally upgraded Kraft paper (TUK), whose properties are presented in Table 1. Both papers were supplied by the same manufacturer and have the same density and thickness (0.2 mm). The polymeric structure of the two papers was essentially the same, since both were obtained through the same manufacturing process. However, additives were added to the TUK paper pulp for the partial neutralization of water-forming agents (melamine, dicyandiamide, and/or polyacrylamide) or for the inhibition of water formation using stabilizing agents (cyanethylation), refs. [11,15]. This provides the TUK paper with greater resistance to ageing by reducing the reaction with water through hydrolysis. 

The following six dielectric liquids were selected as fluids: a non-inhibited mineral oil; three natural esters obtained from sunflower, rapeseed, and soybean; a palm-based modified natural ester; and a synthetic ester. All fluids are commercially available and the main properties, provided by the manufacturers, are given in Table 2. These fluids were selected based on our previous reviewing work, in which it was found that approximately 75% of the available studies analysed these fluids, while the remaining 25% used other fluids or did not specify the types of fluid used. Furthermore, these are recognised as the main commercial esters in some specific reviews, due to their longevity on the market and their popularity, ref. [33].

### 2.2. Accelerated Thermal Ageing

The experimental procedure followed consisted of the following 4 stages: material preparation, drying, impregnation, and thermal ageing. This methodology is used in most ageing studies, such as in [22,25,34]. First, the papers were cut and rolled to fit the dimensions of the 125 mL glass vials. Before being subjected to the ageing temperature, both the fluids and solids were dried, with the aim of replicating the manufacturing process of a transformer and to comply with the threshold values set in the standards. The paper drying was carried out in a forced-air circulation oven for 3 h at 105 °C. The fluids were dried at 60 °C for 24 h in a vacuum oven, applying cycles of 4 h at low pressure (50 mbar) and one hour at medium pressure in an N_2_ atmosphere (500 mbar). The average moisture content of both the fluids and papers after the drying processes is shown in Table 3. The moisture values reached comply with the corresponding regulations, both for the fluids, IEC 60296 [35], IEC 62770 [36], IEC 63012 [37] and IEC 61099 [24], and for the papers, IEEE C57.100 guide [38].

Then, the vials were filled with the corresponding fluid, and copper was introduced, resulting in an oil:paper:copper mass ratio of 16.3:1:1.3 in each vial, following the method defined by [38]. Subsequently, to prevent gas leakage during ageing, the vials were sealed with aluminium caps and butyl-PTFE septa. After that, they were placed in an oven at 60 °C for two hours to ensure the impregnation. Samples identified as 0 h were removed from the oven after completing the impregnation process.

Finally, the oven temperature was raised to reach the ageing temperature. In this study, three different tests were conducted as follows: 130, 150, and 170 °C. The ageing periods in which the samples were analysed were determined based on the solid material used and the applied temperature.

### 2.3. Characterisation

There are different tests to assess the state of cellulose, with one of the most common being the measurement of the DP. This property represents the number of monomers of β-glucose anhydride (C_6_H_10_O_5_) in a cellulose chain. With degradation, the bonds break, reducing the number of monomers.

The measurement of the DP was carried out following the IEC 60450 standard [39], by measuring the viscosity of a paper solution ($v_{s}$) in Bis(ethylenediamine) copper(II) hydroxide (Cu(H_2_NCH_2_CH_2_NH_2_)_2_(OH)_2_). From this value, $v_{s}$, the intrinsic viscosity ($\left[v\right]$) was calculated using Martin’s empirical Formula (1):

(1) $$v_{s}=\left[v\right]\cdot c\cdot 10^{k\cdot \left[v\right]\cdot c}$$

Here, k is the Martin constant (k = 0.14) and c is the concentration of the solution, calculated using the Equation (2):

(2) $$c=\frac{m_{D}}{V_{H_{2}O}+V_{Cu}}$$

where $m_{D}$ is the mass of dry paper added to the solution, $V_{H_{2}O}$ is the volume of added water, and $V_{Cu}$ is the volume of Cu(H_2_NCH_2_CH_2_NH_2_)_2_(OH)_2_.

Finally, the intrinsic viscosity is directly related to the degree of polymerisation by Equation (3):

(3) $$\left[v\right]=K\cdot {\bar{DP}}_{v}^{\alpha }$$

where K and α are the Mark Houwink constants; K = 0.0075 and α = 1, ref. [39].

## 3. Results and Discussion

This section presents the results obtained from the study and provides an assessment based on their analysis. 

### 3.1. DP Evolution during the Ageing

**Figure 1:**
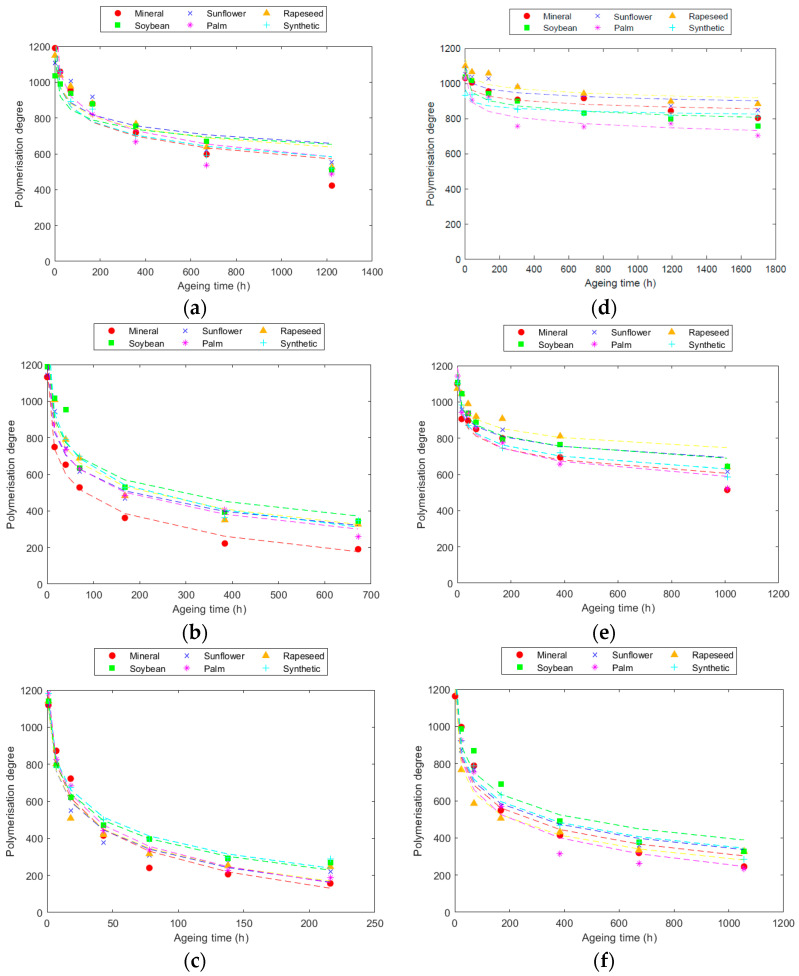
Evolution of the Kraft paper DP at: (**a**) 130 °C; (**b**) 150 °C; and (**c**) 170 °C. Evolution of TUK paper DP at: (**d**) 130 °C; (**e**) 150 °C; and (**f**) 170 °C.

The evolution of the DP of the Kraft and TUK papers with the different fluids at the three temperatures is collected in Figure 1.

The results indicated a rapid decrease in DP at the initial stages of ageing, with a subsequent slowing down of the degradation rate following a logarithmic trend, as was also observed in other works [26]. The degradation process was faster when higher thermal stress conditions were applied to the papers, as was also shown in [23]. Specifically, for the same ageing duration, degradation was multiplied by a factor of 3 for every 20 °C rise in temperature.

In the case of Kraft paper, significant degradation occurred, especially when aged with mineral oil. This degradation was pronounced, reaching end-of-life conditions (DP < 200, ref. [40]) during the tests conducted at 150 °C and 170 °C. Contrasting results were observed in tests with esters, revealing some variations among them. Unmodified natural esters (sunflower, rapeseed, and soybean) demonstrated superior protection of Kraft paper, maintaining a higher DP compared to other fluids at the end of ageing, as was also observed in [8,12,26]. Ageing patterns were remarkably similar for these three fluids at all tested temperatures. Conversely, tests with palm ester showed a substantial drop in the DP compared to other natural esters, with degradation levels approaching those of mineral oil. Synthetic ester, despite providing better protection than mineral oil [13,30,41], performed less effectively than natural esters.

For the TUK paper, the behaviour differed slightly. Unmodified natural esters again proved to be more protective, with the DP decreasing uniformly across all three fluids. Synthetic ester, while resulting in lower PD compared to unmodified natural esters, outperformed palm and mineral fluids, though with a smaller margin than that observed in the Kraft paper. Notably, differences between the two paper types were evident, particularly in the case of palm ester, where TUK paper experienced greater ageing than with mineral oil.

When comparing the degradation of the two papers, TUK paper consistently exhibited a slower degradation rate than Kraft paper across all the analysed cases, as was observed in similar studies [22]. This difference was particularly pronounced during ageing at 130 °C, where degradation was very low. These results clearly demonstrate the protective effect of the additives in TUK paper against degradation, with degradation being approximately 2.5 times lower than that with Kraft paper, due to the reduced hydrolysis.

The results underscore the significant impact of thermal stress on degradation, with slower degradation rates observed at lower temperatures. Additionally, the study revealed that the differences in degradation between various fluids decreased with rising temperatures for both paper types. Specifically, at 170 °C, the advantage of natural esters over mineral oil was diminished compared to the differences observed at 150 °C and 130 °C.

### 3.2. Kinetic Models

Different models allow for the calculation of paper degradation rates as a function of changes in the DP. These models can be used to estimate the time required to reach a certain DP at the analysed temperatures. They are usually based on expressing the number of scissions per monomer, S, as a function of time, (4):

(4) $$S=\frac{1}{DP_{t}}-\frac{1}{DP_{0}}$$

Here, $DP_{t}$ is the DP value at certain time and $DP_{0}$ is the DP value at the initial time.

Each model relates, through different equations, the empirical S values with the temperature-dependent reaction constant, k. From this, the activation energy ($E_{a}$) and the pre-exponential factor (A), specific for each combination of materials, can be calculated through the Arrhenius Equation (5):

(5) $$k=A\cdot \;e^{\frac{-E_{a}}{RT}}$$

where R is the universal gas constant with a value of 8.314 J/mol·K and T is the temperature (K).

The following subsections present different available kinetic models obtained with the results from Section 3.2 in order to determine the best fit to the experimental data

#### 3.2.1. Ekenstam

The simplest model is the zero-order model which, adapted to Equation (6) for high DP values, relates the DP to ageing time through a straight line passing through the origin. This model is based on the work of Ekenstam [42,43], and the Arrhenius equations.

(6) $$\frac{1}{DP_{t}}-\frac{1}{DP_{0}}=k\cdot t$$

Here, t is the ageing time (h). 

**Figure 2:**
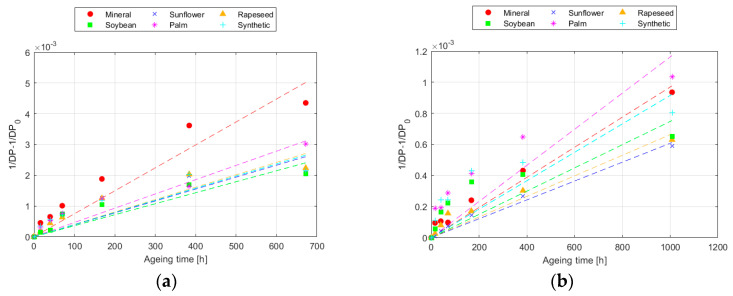
Evolution of the Ekenstam equation at 150 °C for: (**a**) Kraft paper; and (**b**) TUK paper.

The modelling of the DP results following the Ekenstam equation is represented in Figure 2 for 150 °C, whereas the parameters obtained for each temperature are collected in Table 4 and Table 5, for Kraft and TUK paper, respectively. The steeper the slope of the line, the higher the value of k, and the faster the degradation of the paper. Once more, as it was explained in Section 3.1, the higher thermal resistance of the TUK paper can be observed in the figure, since the slope of the TUK paper evolution was smaller than that of the Kraft paper for all the fluids. Therefore, when comparing the k results obtained at different temperatures, it can be seen that k was higher as the temperature increased [41]. When comparing the behaviour of the papers, it was confirmed that the TUK paper aged at a much slower rate than the Kraft paper, with the degradation rate of TUK paper at 170 °C being even lower than that of the Kraft paper at 150 °C.

Moreover, from the value of the reaction constant and the Arrhenius equation, the activation energies required to initiate the reaction of the papers with each of the fluids were obtained. The higher the activation energy, the more energy is required to initiate a specific process [44]. Therefore, applied to the case of thermal ageing, a higher temperature is needed to start the degradation process. The results show that in the ageing process with Kraft paper, the energy required to initiate activation was lower with mineral oil, followed by palm and synthetic esters. The paper impregnated with the unmodified natural esters had the highest activation energies, as was indicated in the DP analysis.

In the case of TUK paper, similar results to those of the Kraft paper were found for the unmodified natural and synthetic esters. However, palm ester required the lowest activation energy. This seems to indicate that if a fluid performs well with a particular type of paper, it does not necessarily mean that it is a good option for another type of paper.

#### 3.2.2. Emsley

The Ekenstam model was modified by Emsley et al. [45] by the Equation (7):

(7) $$\frac{1}{DP_{t}}-\frac{1}{DP_{0}}=\frac{k_{10}}{k_{2}}\cdot (1-e^{{-k}_{2}\cdot t})$$

Here, $k_{10}$ and $k_{2}$ are temperature-dependent parameters. These parameters, related through Equation (8), allow for the calculation of k and how it changes over time:

(8) $$k=k_{10}\cdot e^{-k_{2}\cdot t}$$

**Figure 3:**
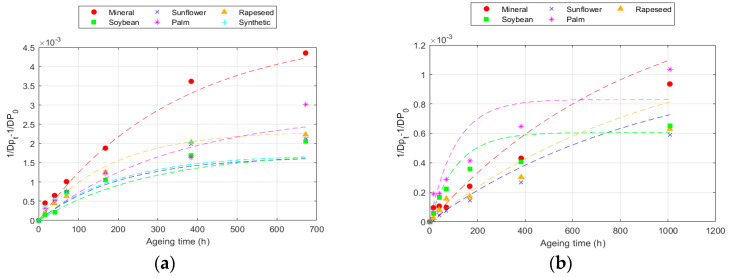
Evolution of the Emsley equation at 150 °C for: (**a**) Kraft paper; and (**b**) TUK paper.

The DP results modelled according to Equation (7) are presented in Figure 3 for 150 °C data and the numerical values for all the cases are collected in Table 6 and Table 7.

As can be observed, the paper degradation is not linear at certain DP values, deviating from the linearity of the zero-model, which implies that the reaction velocity is not constant. This seems to be related to the fact that cellulose materials are not homogeneous, as they are primarily composed of the following three compounds: cellulose, hemicellulose, and lignin. The non-homogeneity causes some compounds to be more reactive than others [34,46].

**Figure 4:**
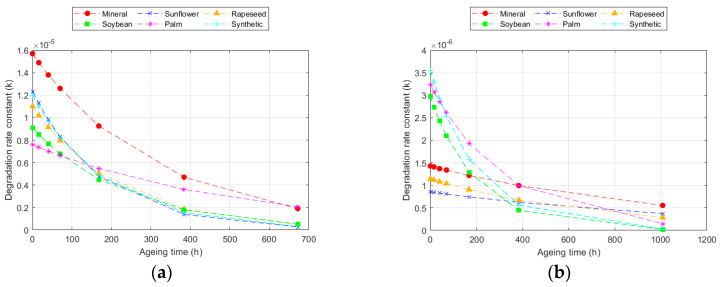
Evolution of the degradation rate at 150 °C according to the Emsley model for: (**a**) Kraft paper; and (**b**) TUK paper.

This model achieved better fits (R^2^) for all temperatures compared to the Ekenstam model, since it adapts to the variation in degradation rate. The values obtained varied, depending on the fluid and paper used. The value of k_10_ increased with the ageing rate. Thus, k_10_ was higher as the temperature increased and was also higher in the Kraft paper than in the TUK one [12,32]. Moreover, k_10_ was higher in the Kraft paper when aged with mineral oil and in the TUK paper when aged with the palm ester. However, in this case, it was necessary to analyse the degradation rate at each point. An example of this calculation is represented in Figure 4, which shows the degradation data for the two types of cellulose materials at 150 °C. The ageing rate changed over time, being higher in the early stages and slowing down later. This rate changed depending on the paper and fluid used; for the same exposure time at the same temperature, it was lower in the TUK paper. While the degradation rate decreased as the exposure time increased, the change was not the same in all the fluids. Some of them, such as the palm ester, showed a more constant ageing rate, while others, such as the sunflower ester, had a greater change [31,47].

#### 3.2.3. Zervos

In addition to the previous models, some authors have subsequently proposed equations to characterise specific types of paper. In 2005, Zervos and Moropoulou [48] defined a specific kinetic model, (9), for cotton cellulose when aged in closed systems:

(9) $$\left(\frac{1}{DP_{t}}-\frac{1}{DP_{0}}\right)\cdot 100=a\cdot (2^{k\cdot t}-1)$$

Here, a is a temperature-dependent constant. 

**Figure 5:**
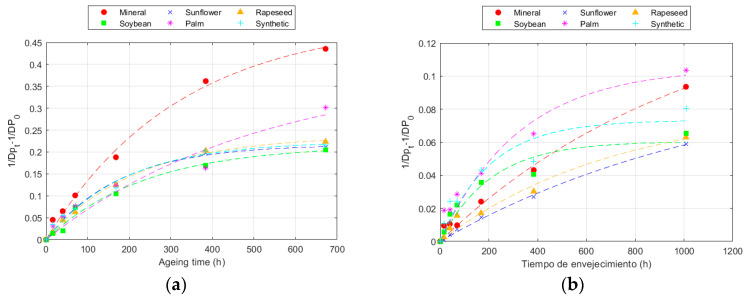
Evolution of the Zervos equation at 150 °C for: (**a**) Kraft paper; and (**b**) TUK paper.

The fit of the DP variation according to this model is shown in Figure 5 for the 150 °C results, whereas the parameters k for all the cases are collected in Table 8 and Table 9.

This model, despite being specifically developed for cotton material, allowed for a good fit of the degradation of the two papers studied in this work and the fit was similar to that of the Emsley model.

#### 3.2.4. Calvini

In 2006, Calvini and Gorassini [49] proposed a new interpretation of kinetic models. Until then, all models were based on expressing the number of scissions per monomer as a function of time (4). Instead, this model proposes the relationship with scissions per chain, $S_{c}$, (10):

(10) $$S_{c}=\frac{DP_{0}}{DP_{t}}-1$$

Based on this parameter, they suggested the expression (11):

(11) $$\left(\frac{DP_{0}}{DP_{t}}-1\right)=\left(\frac{DP_{0}}{LODP}\right)\cdot (1-e^{-k\cdot t})$$

This model considers the levelling-off degree of polymerisation (LODP), corresponding to a third state of degradation, where degradation occurs very slowly, ref. [3]. This state is only found in drastic degradations below a certain DP value, where the mechanical strength of cellulose is completely lost. Before reaching this state, cellulose polymers pass through two different states. In the first state, the weakest points are attacked, leading to a sharp decrease in DP. In the second state, where the amorphous fraction is lost, the decrease is slower [41]. Therefore, to apply this model, it is necessary to set an end-of-life criterion, which is usually a DP of 200 [40], but this depends on the paper and fluid analysed [12].

**Figure 6:**
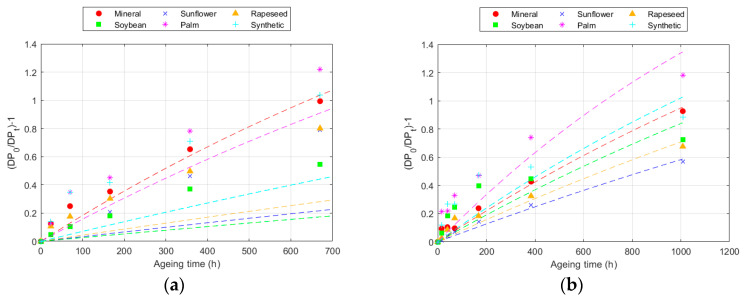
Evolution of the Calvini equation at 150 °C for: (**a**) Kraft paper; and (**b**) TUK paper.

The variations in the DP analysed through this model are presented in Figure 6 for the 150 °C tests, whereas the numerical results for all the temperatures are shown in Table 10 and Table 11.

The LODP was limited to a DP of 400 due to convergence limits. As shown in the results, ageing at 130 °C did not allow for significant degradation values to be reached in any of the papers studied. The lack of data for values close to the DP limit hindered the development of the model with a good fit, as can be seen in the results. For the lower temperature, the fit was very low for unmodified natural esters, and was acceptable for other temperatures, though lower than in other models. In the case of the TUK paper, the fit was better for all fluids compared to the Kraft paper, as can also be inferred from the figure, except for the synthetic ester, a fluid for which this model should not be applied.

Despite the lower fit compared to other models, the constant *k* allows for an assessment of the degradation rate with each fluid, leading to conclusions similar to those obtained with other models. The lower values of the reaction rates of the TUK paper in comparison with the Kraft ones agrees with the higher thermal resistance of this paper, as explained in previous sections. However, this model is highly dependent on the LODP, as well as on the available data. In any case, other models provided better fits when data for a very low DP were not available.

### 3.3. Estimation of Remaining Life

The described models allow for the evaluation of degradation based on the reaction rate represented by the constant k and the activation energy required for the degradation reaction to occur. Although this allows for the comparison of different dielectric fluids and cellulose materials, it does not provide a direct estimate of the expected transformer life. For this purpose, the Institute of Electrical and Electronics Engineers (IEEE) proposes an expression, as shown in Equation (12), for calculating the remaining life based on temperature [17]:

(12) $$L\left(T\right)=A\cdot e^{B/T}$$

where A and B are constants depending on the degradation rate, T is the temperature (K), and L(T) is the time required to reach the set DP end-of-life value (LODP).

The results of the experiment were used to obtain the parameters of the IEEE model. In order to directly use experimental data, the constants were defined for a DP of 550, as this was the lowest DP achieved during the ageing at 130 °C.

Additionally, it was decided that the first-order model should be used, due to its good fit, to estimate the time required to reach a lower DP (350) at each temperature. Previously, the accuracy of the time calculated with this model was validated, as shown in Table 12 and Table 13. The results were notably similar for the highest temperature, where there were sufficient end-of-life data for the paper. At the temperature of 130 °C, the model did not adapt as precisely to reality. At this temperature, the LODP was significantly high, around 500 for Kraft paper and 700 for TUK paper, as was found in other studies [12,22].

**Figure 7:**
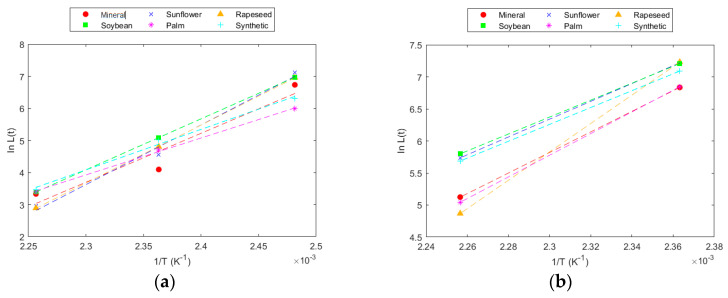
IEEE model for DP = 550 in: (**a**) Kraft paper; and (**b**) TUK paper.

From these values, the parameters of the IEEE model were obtained for each fluid and paper. The curves derived from the IEEE model are presented in Figure 7, whereas the parameters obtained using experimental data and those derived from the Emsley model are collected in Table 14 and Table 15.

This model allows for the direct calculation of the time required to reach the fixed DP at any temperature. The model fit was very high, and the parameters obtained for the experimental data were very similar to those obtained with data calculated from the Emsley model.

According to the experimental data obtained in this work, under the analysed conditions, a DP of 200 at 130 °C would be reached at very distant time points. The data trend showed an asymptote that causes this time to tend towards infinity. This behaviour was reflected in the kinetic models, indicating that it is not possible to reach such a low DP at that temperature. Similarly, in the case of the TUK paper, with the data available in this work, it would not be possible to reach a DP of 550, as seen in the DP results, where the degradation slope was very small, and this value would be reached at very distant times. These results deviate from reality because, in an operating transformer, there are more factors contributing to degradation, such as electrical and mechanical stress and the presence of other materials, in addition to certain faults that may occur in the equipment and can affect solid insulation. In the test carried out, in a controlled atmosphere and with the system subjected only to thermal stress, degradation stabilized in a way that the time required to reach lower levels of ageing was very large.

Based on these parameters, it would be possible to estimate the life expectancy, or the time required to reach a certain DP at different temperatures in each insulation system. The results obtained, collected in Table 16, are an approximation for conditions similar to those replicated in this experiment. Consistent with previous findings, the TUK paper exhibits greater resistance to aging, attributed to the lower hydrolysis occurring within it, thereby reducing the aging rate, and prolonging the time it takes to reach a certain DP compared to the Kraft paper.

## 4. Conclusions

The thermal degradation of cellulose polymers in Kraft and TUK papers was analysed when combined with five different alternative fluids and a mineral oil through the measurement of the evolution of their polymerisation degree.

From these results, it was found that cellulose ageing differed from one fluid to another. For both types of paper, sunflower, rapeseed, and soybean esters demonstrated superior performance, with no notable differences among them concerning the papers’ DP. Synthetic ester, on the other hand, offered intermediate levels of protection, which were always better than those of the mineral oil. However, the performance of palm ester varied depending on the cellulosic material. This fluid protected the Kraft paper better than the mineral oil, but performed the worst with the TUK paper.

Moreover, the effect of the additives in the TUK paper on its thermal degradation was noticeable, since its DP reduction was up to 2.5 times lower than that of the Kraft paper, under all conditions.

Regarding the kinetic models, five different alternatives were obtained with the DP data. The resulting parameters met the trends observed in the gross analysis of the degradation. Moreover, the results showed that the Emsley model was a better fit for the materials studied in this work, probably due to the flexibility of the equations and the consideration of the non-linearity of the ageing rate.

Based on this, it is not only possible to confirm the trends observed in other previous works, but also state that unmodified natural esters are always the best option in terms of paper protection, without significant differences being observed among them. Furthermore, due to the wide variety of materials analysed, following a unique methodology, the obtained models allow for comparisons with many different combinations. This makes them a suitable tool for verifying, in each operating condition, the advantages of choosing TUK paper and enabling the selection of the best option among the different esters available on the market.

## Figures and Tables

**Table 1:** Properties of the analysed papers.

Property	Kraft	TUK
Apparent density [g/cm^3^]	1	1
Tensile strength unfolded (machine direction) [MPa]	94	115
Elongation unfolded (machine direction) [%]	1.7	2
Moisture content [%]	<8	<8
Ash content [%]	0.3	0.3
Conductivity of aqueous extract [mS/m]	2	2.2
Electric strength in air unfolded [kV/mm]	10	10

**Table 2:** Properties of the studied commercial fluids.

Property	Mineral	Sunflower	Rapeseed	Soybean	Palm	Synthetic
Density 20 °C [g/cm^3^]	0.839	0.91	0.92	0.92	0.97	0.839
Kinematic viscosity 40 °C [mm^2^/s]	9.98	39.2	37	34	5.062	29
Flash point [°C]	176	330	>315	320	188	260
Fire point [°C]	-	362	>350	350	206	316
Pour point [°C]	−48	−25	−31	−18	−37.5	−56
Acidity [mg KOH/g]	<0.01	0.05	≤0.04	<0.05	<0.01	<0.03
Moisture [ppm]	15	150	50	4–50	52	50
Dielectric dissipation factor 90 °C	0.002	0.03	<0.03	<0.03	<0.003	<0.008
Breakdown voltage [kV]	46	65	>75	≥55	85	>75
Biodegradability	-	85	98	>99	77	89

**Table 3:** Average moisture content of the insulating materials at the beginning of the thermal ageing.

Moisture	Mineral	Sunflower	Rapeseed	Soybean	Palm	Synthetic
Fluid [ppm]	16.0	64.4	59.5	46.8	56.3	114.7
Kraft [%]	0.5	0.3	0.4	0.4	0.5	0.5
TUK [%]	0.5	0.4	0.5	0.4	0.5	0.5

**Table 4:** Degradation rate and energy activation of Kraft paper according to the Ekenstam model.

Fluid	130 °C		150 °C		170 °C		Ea (×10^5^) (J/mol)	A (×10^15^) (h^−1^)
	k (×10^−8^)	R^2^	k (×10^−6^)	R^2^	k (×10^−5^)	R^2^		
Mineral	20.00	0.82	7.00	0.95	3.00	0.97	1.87	480
Sunflower	9.00	0.70	4.00	0.89	2.00	0.91	2.02	17,000
Rapeseed	10.00	0.75	4.00	0.92	2.00	0.99	1.98	5700
Soybean	8.00	0.67	4.00	0.93	2.00	0.94	2.06	58,000
Palm	20.00	0.79	5.00	0.96	2.00	0.98	1.87	4.90
Synthetic	10.00	0.76	4.00	0.92	1.00	0.93	1.88	3.36

**Table 5:** Degradation rate and energy activation of TUK paper according to the Ekenstam model.

Fluid	130 °C		150 °C		170 °C		Ea (×10^5^) (J/mol)	A (×10^16^) (h^−1^)
	k (×10^−8^)	R^2^	k (×10^−7^)	R^2^	k (×10^−6^)	R^2^		
Mineral	2.00	0.94	10.00	0.98	3.00	0.98	1.88	6.05
Sunflower	1.00	0.98	6.00	0.99	3.00	0.93	2.13	5810
Rapeseed	1.00	0.95	7.00	0.96	3.00	0.97	2.13	6440
Soybean	1.00	0.92	7.00	0.94	2.00	0.97	1.98	83
Palm	3.00	0.88	10.00	0.98	4.00	0.95	1.83	1.96
Synthetic	2.00	0.80	9.00	0.84	3.00	0.98	1.87	5.64

**Table 6:** Parameters of Kraft paper according to the Emsley model.

Fluid	130 °C			150 °C			170 °C		
	k_10_ (×10^−6^)	k_2_ (×10^−3^)	R^2^	k_10_ (×10^−5^)	k_2_ (×10^−3^)	R^2^	k_10_ (×10^−5^)	k_2_ (×10^−3^)	R^2^
Mineral	2.10	1.20	0.98	1.60	3.10	0.99	4.50	6.00	0.98
Sunflower	1.70	1.60	0.99	1.20	5.70	1.00	5.10	14.00	0.98
Rapeseed	1.70	1.50	0.99	1.10	4.70	1.00	5.30	16.00	0.98
Soybean	1.50	1.50	0.95	0.91	4.20	0.99	3.60	12.00	0.98
Palm	3.00	1.80	0.99	0.76	1.90	0.94	3.60	5.60	0.99
Synthetic	2.70	1.80	0.98	1.20	5.30	0.99	3.70	1.3.0	0.99

**Table 7:** Parameters of TUK paper according to the Emsley model.

Fluid	130 °C			150 °C			170 °C		
	k_10_ (×10^−6^)	k_2_ (×10^−3^)	R^2^	k_10_ (×10^−6^)	k_2_ (×10^−3^)	R^2^	k_10_ (×10^−6^)	k_2_ (×10^−3^)	R^2^
Mineral	0.36	1.30	0.95	1.40	0.94	0.98	5.10	1.20	0.99
Sunflower	0.29	1.10	0.99	0.86	0.83	1.00	6.10	2.50	0.97
Rapeseed	0.83	1.30	0.98	1.10	1.40	0.96	11.00	4.90	0.92
Soybean	1.00	3.10	0.87	3.00	4.90	0.92	4.40	1.60	1.00
Palm	1.20	3.10	0.88	3.20	3.10	0.94	8.30	2.10	0.99
Synthetic	1.70	6.30	0.93	3.50	4.80	0.88	4.40	1.20	0.98

**Table 8:** Parameters of Kraft paper according to Zervos model.

Fluid	130 °C			150 °C			170 °C		
	a (×10^−1^)	k (×10^−3^)	R^2^	a (×10^−1^)	k (×10^−3^)	R^2^	a (×10^−1^)	k (×10^−2^)	R^2^
Mineral	−1.70	−1.80	0.98	−5.00	−4.60	0.99	−7.50	−0.87	0.99
Sunflower	−1.00	−2.40	0.99	−2.20	−8.20	1.00	−3.60	−2.00	0.99
Rapeseed	−1.10	−2.20	0.99	−2.30	−6.30	1.00	−3.30	−2.30	0.99
Soybean	−0.96	−2.20	0.95	−2.20	−5.80	0.99	−3.10	−1.70	0.99
Palm	−1.70	−2.60	0.99	−4.20	−2.40	0.94	−6.40	−0.81	1.00
Synthetic	−1.40	−2.70	0.98	−2.20	−6.30	0.99	−2.90	−1.80	0.99

**Table 9:** Parameters of TUK paper according to Zervos model.

Fluid	130 °C			150 °C			170 °C		
	a (×10^−2^)	k (×10^−3^)	R^2^	a (×10^−1^)	k (×10^−3^)	R^2^	a (×10^−1^)	k (×10^−3^)	R^2^
Mineral	−2.90	−1.80	0.91	−1.50	−1.40	0.99	−4.40	−1.70	0.99
Sunflower	−3.20	−1.00	0.99	−1.00	−1.20	1.00	−2.40	−3.70	0.97
Rapeseed	−2.40	−2.10	0.99	9.70	0.95	0.92	−2.20	−7.00	0.94
Soybean	−4.10	−2.80	0.95	−0.60	−7.10	0.93	−2.70	−2.30	1.00
Palm	−4.30	−3.80	0.91	−1.10	−4.40	0.95	−4.00	−3.00	0.99
Synthetic	−2.70	−8.80	0.95	−0.74	−6.80	0.90	−3.30	−0.01	0.94

**Table 10:** Parameters of Kraft paper according to the Calvini model.

Fluid	130 °C		150 °C		170 °C	
	k (×10^−4^)	R^2^	k (×10^−3^)	R^2^	k (×10^−2^)	R^2^
Mineral	5.10	0.88	9.10	0.74	2.98	0.57
Sunflower	0.55	0.37	3.74	0.97	2.78	0.89
Rapeseed	0.74	0.56	3.50	0.99	2.62	0.92
Soybean	0.51	0.20	2.68	0.97	1.64	0.97
Palm	9.71	0.88	3.80	0.91	2.30	0.73
Synthetic	6.57	0.72	2.94	0.98	1.49	0.98

**Table 11:** Parameters of TUK paper according to the Calvini model.

Fluid	130 °C		150 °C		170 °C	
	k (×10^−5^)	R^2^	k (×10^−4^)	R^2^	k (×10^−3^)	R^2^
Mineral	6.33	0.84	3.80	0.98	2.99	0.93
Sunflower	5.51	0.96	2.16	0.99	2.45	0.97
Rapeseed	5.96	0.87	2.69	0.94	3.57	0.92
Soybean	11.30	0.77	3.28	0.64	1.94	0.99
Palm	10.70	0.64	5.91	0.81	4.79	0.85
Synthetic	7.34	0.14	4.15	0.63	2.41	0.95

**Table 12:** Time (h) to DP 550 in the Kraft paper according to the experimental data and Emsley model.

Fluid	130 °C		150 °C		170 °C	
	Exp.	Emsley	Exp.	Emsley	Exp.	Emsley
Mineral	840	682	60	66	28	22
Sunflower	1230	1440	95	100	19	21
Rapeseed	1050	1166	122	117	18	21
Soybean	1070	1458	160	143	30	31
Palm	400	373	110	151	30	29
Synthetic	550	497	150	126	32	32

**Table 13:** Time (h) to DP 550 in the TUK paper according to the experimental data and Emsley model.

Fluid	150 °C		170 °C	
	Exp.	Emsley	Exp.	Emsley
Mineral	930	833	168	225
Sunflower	1365	1700	310	214
Rapeseed	1380	1906	130	122
Soybean	1350	1532	330	285
Palm	940	1180	155	140
Synthetic	1200	1618	295	268

**Table 14:** Parameters of Kraft paper according to the IEEE model.

Fluid	Experimental (DP 550)			Emsley (DP 550)			Emsley (DP 350)	
	A (×10^−16^)	B (×10^4^)	R^2^	A (×10^−16^)	B (×10^4^)	R^2^	A (×10^−16^)	B (×10^4^)
Mineral	210.00	1.50	0.92	150.00	1.50	0.97	21,000,000	1.10
Sunflower	0.08	1.90	0.99	0.05	1.90	0.98	0.12	1.90
Rapeseed	0.29	1.80	1.00	0.49	1.80	1.00	0.31	1.90
Soybean	68.00	1.60	0.99	3.60	1.70	0.99	0.31	1.90
Palm	1,500,000	1.20	0.99	2,600,000	1.10	0.96	130.00	1.60
Synthetic	130,000	1.30	0.99	330,000	1.20	1.00	17.00	1.70

**Table 15:** Parameters of TUK paper according to the IEEE model.

Fluid	Experimental (DP 550)		Emsley (DP 550)	
	A (×10^−16^)	B (×10^4^)	A (×10^−16^)	B (×10^4^)
Mineral	340.00	1.60	1,000,000	1.20
Sunflower	51,000	1.40	0.31	1.90
Rapeseed	0.0003	2.20	0.0001	2.60
Soybean	38,000	1.30	940.00	1.60
Palm	47.00	1.70	0.04	2.00
Synthetic	38,000	1.30	130.00	1.70

**Table 16:** Time (days) to DP 550 at different temperatures.

Fluid	70 °C		90 °C		110 °C	
	Kraft	TUK	Kraft	TUK	Kraft	TUK
Mineral	8600	256,982	773	19,666	89	1968
Sunflower	399,206	1,473,259	18,868	69,633	1227	4527
Rapeseed	74,671	8,665,817	4144	252,967	311	10,681
Soybean	51,396	710,479	3933	54,372	394	5442
Palm	9769	655,671	1421	42,731	253	3704
Synthetic	15,627	1,813,558	1936	118,193	298	10,245

## Data Availability

Data are contained within the article.
